# Forum COVID-19: Geistes- und sozialwissenschaftliche Perspektiven

**DOI:** 10.1007/s00048-020-00251-x

**Published:** 2020-05-11

**Authors:** Karen Nolte

**Affiliations:** Institut für Geschichte und Ethik der Medizin, Ruprecht-Karls-Universität Heidelberg, Im Neuenheimer Feld 327, 69120 Heidelberg, Deutschland

Der Historiker Malte Thießen hat vor wenigen Jahren herausgestellt, dass sich gegenwärtige Gesellschaften im „Zeitalter der Immunität“ wähnten – diese Gewissheit einer „Immunisierten Gesellschaft“ hat nun durch COVID-19 Risse bekommen (Thießen 2017). Medizinhistorische Expertise ist derzeit zur Bewertung des gesellschaftlichen und politischen Umgangs mit der COVID-19-Pandemie in den populären Medien sehr gefragt. Vor COVID-19 folgte die öffentliche Darstellung und Wahrnehmung des klassischen medizinhistorischen Forschungsgebiets „Seuchengeschichte“ eher einem Fortschrittsnarrativ, demzufolge Menschen in vergangenen Gesellschaften gefährlichen Infektionskrankheiten schutzlos ausgeliefert waren, und die wiederkehrende Gefahr der Epi- und Pandemien dank des Siegeszugs der Bakteriologie im 20. Jahrhundert endgültig überwunden werden konnte. Nun wird der Auseinandersetzung mit historischen „Seuchen“, vor allem mit der Influenza-Pandemie von 1918–1920, der „Spanischen Grippe“, eine andere Funktion zugeschrieben: Aus der Geschichte sollen Politik und Gesellschaft Lehren für die aktuellen Herausforderungen der Pandemie ziehen. In dem Bewusstsein, selbst inmitten eines Pandemiegeschehens historischen Ausmaßes zu stehen, erhält die medizin- und wissenschaftsgeschichtliche Forschung über Epi- und Pandemien derzeit ebenso kräftigen Aufwind wie sozial- und kulturwissenschaftliche Analysen von Krisen und Katastrophen.

Für den Auftakt dieses Forums wurden daher Wissenschaftler*innen des skizzierten Forschungsfelds angefragt, ihre Expertise einzubringen und ihr Wissensgebiet vor dem Hintergrund von Beobachtungen des aktuellen gesellschaftlichen, politischen und wissenschaftlichen Umgangs mit der COVID-19-Pandemie in den letzten Wochen zu reflektieren sowie aktuelle Fragestellungen und Thesen zur Seuchen- bzw. Pandemiegeschichte herauszuarbeiten. Auch soll es darum gehen, jenseits der derzeitigen Leitwissenschaft Virologie die gegenwärtige Situation einer wissenschaftlichen Analyse aus Sicht einer geistes- und sozialwissenschaftlich orientierten Wissenschaftsforschung zu unterziehen.

Mit den folgenden Beiträgen eröffnet NTM die wissenschaftliche Diskussion rund um COVID-19 und fordert dazu auf, weitere Beiträge aus Sicht von Medizin‑, Wissenschafts- und Technikgeschichte und STS zum Thema (max. 15.000 Zeichen incl. Leerzeichen und Literatur) einzureichen.
